# NegFluo, a Fast and Efficient Method to Determine Starch Granule Size and Morphology *In Situ* in Plant Chloroplasts

**DOI:** 10.3389/fpls.2019.01075

**Published:** 2019-09-09

**Authors:** Camille Vandromme, Angelina Kasprowicz, Adeline Courseaux, Dave Trinel, Maud Facon, Jean-Luc Putaux, Christophe D’Hulst, Fabrice Wattebled, Corentin Spriet

**Affiliations:** ^1^Univ. Lille, CNRS, UMR8576 – UGSF – Unité de Glycobiologie Structurale et Fonctionnelle, Lille, France; ^2^Univ. Grenoble Alpes, CNRS, CERMAV, Grenoble, France

**Keywords:** starch, confocal fluorescence imaging, machine learning, *Arabidopsis*, starch granule morphology, autofluorescence

## Abstract

Starch granules that accumulate in the plastids of plants vary in size, shape, phosphate, or protein content according to their botanical origin. Depending on their size, the applications in food and nonfood industries differ. Being able to master starch granule size for a specific plant, without alteration of other characteristics (phosphate content, protein content, etc.), is challenging. The development of a simple and effective screening method to determine the size and shape of starch granules in a plant population is therefore of prime interest. In this study, we propose a new method, NegFluo, that combines negative confocal autofluorescence imaging in leaf and machine learning (ML)-based image analysis. It provides a fast, automated, and easy-to-use pipeline for both *in situ* starch granule imaging and its morphological analysis. NegFluo was applied to *Arabidopsis* leaves of wild-type and *ss4* mutant plants. We validated its accuracy by comparing morphological quantifications using NegFluo and state-of-the-art methods relying either on starch granule purification or on preparation-intensive electron microscopy combined with manual image analysis. NegFluo thus opens the way to fast *in situ* analysis of starch granules.

## Introduction

Starch is a polysaccharide that accumulates in plants and is synthesized in either photosynthetic or storage organs. In leaves, starch accumulates during the day and is degraded at night to provide carbon and energy for the plant in the absence of photosynthetic activity (Pfister and Zeeman, 2016). The polysaccharide synthetized in seeds or tubers represents the main source of caloric intake of human nutrition. Millions of tons of starch are extracted each year in the world from three main species: maize, wheat, and potato (Burrell, 2003). Depending on the plant source, the shape, size, and composition of the starch granule (amylose/amylopectin ratio, but also lipid, protein, or phosphate content) vary greatly (Jane et al., 1994). This is why the biological origin of starch strongly influences the properties and use of the polymer (Singh et al., 2003; Singh et al., 2007; Alcazar-Alay and Meireles, 2015). A large part of the starch extracted is enzymatically digested to produce sweeteners. It is also often chemically or physically modified to impart new properties. The starch granule size is one of the parameters that influence its digestibility (Dhital et al., 2010; Abebe et al., 2015) and the effectiveness of chemical and physical transformations (Zhao et al., 2015; Wang et al., 2016; Farooq et al., 2018). In this context, controlling the factors conditioning starch granule size *in planta* without impairing neither the ultrastructure nor the composition of the granules would represent a decisive advantage. Understanding the initial steps of starch synthesis would open the route to control the number of starch granules produced in each plastid and consequently their size for a given carbon flux: the fewer initiation events, the larger the granules.

In recent years, various factors involved in new starch granule initiation and influencing the size and/or shape of these granules have been identified in *Arabidopsis thaliana* (Malinova et al., 2018). These proteins include specific isoforms of starch synthases (SSs), SS4 and SS3 (Roldán et al., 2007; Szydlowski et al., 2009), as well as non-catalytic proteins such as PTST2 and PTST3 (Seung et al., 2017), PII1/MRC, and MFP1 (Seung et al., 2018; Vandromme et al., 2019).

Starch synthesis in the photosynthetic organs is very similar to that in the storage organs and requires the same set of proteins (Pfister and Zeeman, 2016). Moreover, genes coding proteins involved in the starch initiation process in *Arabidopsis* are also conserved in crops. Therefore, increasing our knowledge of the factors influencing the size and/or shape of starch granules on a model species will provide new possibilities for varietal improvement.

Nevertheless, the acquisition of this knowledge requires the establishment of a simple and rapid screening procedure. Current tools for the detection of change in size and shape of starch granules require the extraction and purification of the granules, followed by a particle counteranalysis (Vandromme et al., 2019) or by sample observations under a light or electron microscope (Roldán et al., 2007; Lappe et al., 2017). In addition, variations in the number of starch granules per plastid are generally evaluated from leaf sections observed under a light or electron microscope. This technique, however, depends on the section plane, and a large number of analyses must be carried out to obtain statistically reliable data (Seung et al., 2017). While these different techniques are suitable to characterize a specific mutant line, they are cumbersome, expensive, and ultimately incompatible for screening large collection of individuals.

In this paper, we describe an image acquisition and analysis method that requires only a minimum of preparation and yet sufficiently resolutive to identify a variation in the size and shape of starch granules in plant tissues. Furthermore, the proposed method is easy to handle without any prior knowledge in programming or ML and only requires a traditional confocal microscope. We focused our study on starch accumulated in the chloroplasts of leaf cells of the model species *A. thaliana*. In a wild-type line, chloroplasts contain on average five to seven starch granules having a size between 0.8 and 1 µm (Malinova et al., 2018; Vandromme et al., 2019). Our technique was also applied to a mutant line impaired for the soluble SS4. This line exhibits a reduced number of starch granules per chloroplast. Moreover, the remaining granules, compared with the wild type, are modified in size and shape, conferring a good validation of the new screening method (Roldán et al., 2007).

## Materials and Methods

### Plant Material and Growth Conditions

*A. thaliana* lines were obtained from Nottingham Arabidopsis Stock Centre [NASC; http://arabidopsis.info (Alonso et al., 2003)]. Columbia (Col-0) line was used as wild-type reference, and an SS4-deficient line corresponds to *ss4-1* (GABI_290D11) already described in Roldán et al. (2007). In all cases, plants were grown in a growth chamber (GroBank, BB-XXL.3+). Conditions used were 16 h : 8 h, light : dark photoperiod at 23°C during the day and 20°C during the night, and 120 µmol photon m^−2^ s^−1^. Seeds were incubated at 4°C in 0.1% Phytagel solution (w/v) for 3 days before being sown on peat-based compost.

### Sample Preparation

Sample preparation was adapted from the protocol described in Vandromme et al. (2019). For each genotype, one leaf of 2-week-old plants was harvested at the end of the light phase and placed under vacuum in 1 mL of fixating solution (4% (w/v) paraformaldehyde, 4% (w/v) sucrose, and phosphate-buffered saline (PBS 1×) at pH 7.3). Fixed leaves were washed in water. A section of 2 × 2 mm was then placed between a slide and a coverslip. To ensure optimal acquisition, the leaf midribs were excluded from the selected samples from which the lower epidermis is placed in front of the objective lens.

### Confocal Acquisition

The prepared samples were observed under A1 Nikon confocal microscope (Nikon Instruments Europe B.V.) with a Plan Apo 60x Oil (NA = 1.4) objective. The autofluorescence was acquired with λ_ex_ = 488 nm and λ_em_ = 500–550 nm (green channel) and with λ_ex_ = 561 nm and λ_em_ = 570–620 nm (red channel). To further increase the contrast between leaf autofluorescence and starch darkness, median filtering and contrast-limited adaptative histogram equalization (block size 20, histogram bins 50, maximum slope 2.5) was applied using an ImageJ macro before the ML step (Majunder and Kumar, 2014).

### Machine Learning

The ML approach is based on the “Waikato Environment for Knowledge Analysis” (WEKA) implemented in ImageJ (Witten et al., 2017). We first defined a classification between four categories: starch granule, plastid, membranes, and background. These training features thus provided more than 300 parameters for each pixel and allowed unambiguous discrimination between the four classes after appropriate training of a random forest classifier with 200 initial trees. Manual labeling was performed in a pixel-by-pixel basis between the four classes. To avoid overlearning, training was performed on confocal images of leaves from the wild type and a mutant line affected for the starch metabolism. Each image is then segmented based on obtained probability maps (see method description for segmentation) and compared with results achieved through manual segmentation. Once 80% of starch granules are correctly detected by NegFluo-ML, the classifier is applied to new sets of images to validate the training step, and similar results were obtained (more than 80% correct detection). Then, for each initial image, a probability map was obtained for each category and was used for segmentation.

### Segmentation

Automated analysis allows screening through a high number of images. Thus, we chose to analyze starch granules with the highest confidence level possible. Probability maps were first thresholded to an 80% confidence interval in each pixel for both starch granules and plastids. To reinforce the accuracy, we developed an ImageJ macro combining (i) various binary operations to ensure that detected starch granules were completely embedded into plastids, (ii) watershed operations to separate adjacent granules (Beucher, 1992), and (iii) automated particle detection, setting in ImageJ (1) “Set Measurements” to area, perimeter, and fit ellipses (from which diameter and major and minor axes are extracted) and (2) applying the “analyze particles” module with *in situ* visualization of segmented starch granules. These three additional pipeline steps ensure that starch granules that are out of focus or at the edge of the image are excluded from statistical analysis. From extracted parameters, mean values and standard errors were calculated and plotted using Excel (Microsoft). Standard error does not represent a measurement error but corresponds to the diversity of starch granules size.

### Granule Size Distribution

The starch purification and granule size determination using a Multisizer 4 Coulter counter (Beckman Coulter Life Science, Indianapolis, IN, USA) equipped with a 20-mm aperture tube are fully described inVandromme et al. (2019).

### Scanning Electron Microscopy

Leaf fragments were fixed with glutaraldehyde, post-fixed with osmium tetroxide (OsO_4_), and embedded in Epon resin. The blocks were cut with a diamond knife in a Leica UC6 ultramicrotome. Backscattered electron images of the non-conductive surface were recorded under a low pressure of air (100 Pa) in a FEI Quanta 250 FEG environmental scanning electron microscope (ESEM) operating at 7 kV.

### Transmission Electron Microscopy

Samples were prepared as described in Boyer et al. (2016). Leaves from 3-week-old plants were harvested at the end of the day and fixed with glutaraldehyde. Samples were post-fixed with OsO_4_ and embedded in Epon resin. About 70-nm-thin sections were cut with a diamond knife in a Leica UC6 ultramicrotome and post-stained with periodic acid–thiosemicarbazide–silver proteinate (PATAg) (Gallant and Guilbot 1969). Samples were observed with a Philips CM200 transmission electron microscope (TEM) operating at 80 kV. Images were recorded on Kodak SO163 films.

## Results

### *In Situ* Confocal Starch Imaging

*In situ* starch granule imaging performed on fresh leaves is quite challenging. Indeed, most plastid components generate a high autofluorescence with a global broad excitation and emission spectrum. Thus, traditional strategies involving tagging, like iodine staining (Ovecka et al., 2012), are not contrasted enough and thus not efficient. In this study, we decided to invert the problem. Indeed, starch granules, contrary to all the other leaf cell components, present a low autofluorescence. Thus, we decided to develop an approach based on inverse fluorescence imaging of starch granule (NegFluo). The first step was to perform the spectral characterization of the leaves.

Our spectral observations of *A. thaliana* leaves allowed us, according to the literature (Roshchina, 2012), to determine three main spectral emission bands of autofluorescence: (i) in blue for a maximum emission at 450 nm (azulenes and phenols) and (ii) in green, for a maximum at 550 nm (carotenoids), and in red, for a maximum at 680 nm (chlorophyll). A more precise analysis of the differential between the “non-signal” of the starch granules and the surrounding signals allowed us to limit the procedure to a two-channel focusing on carotenoids and chlorophyll.

**Figure 1:**
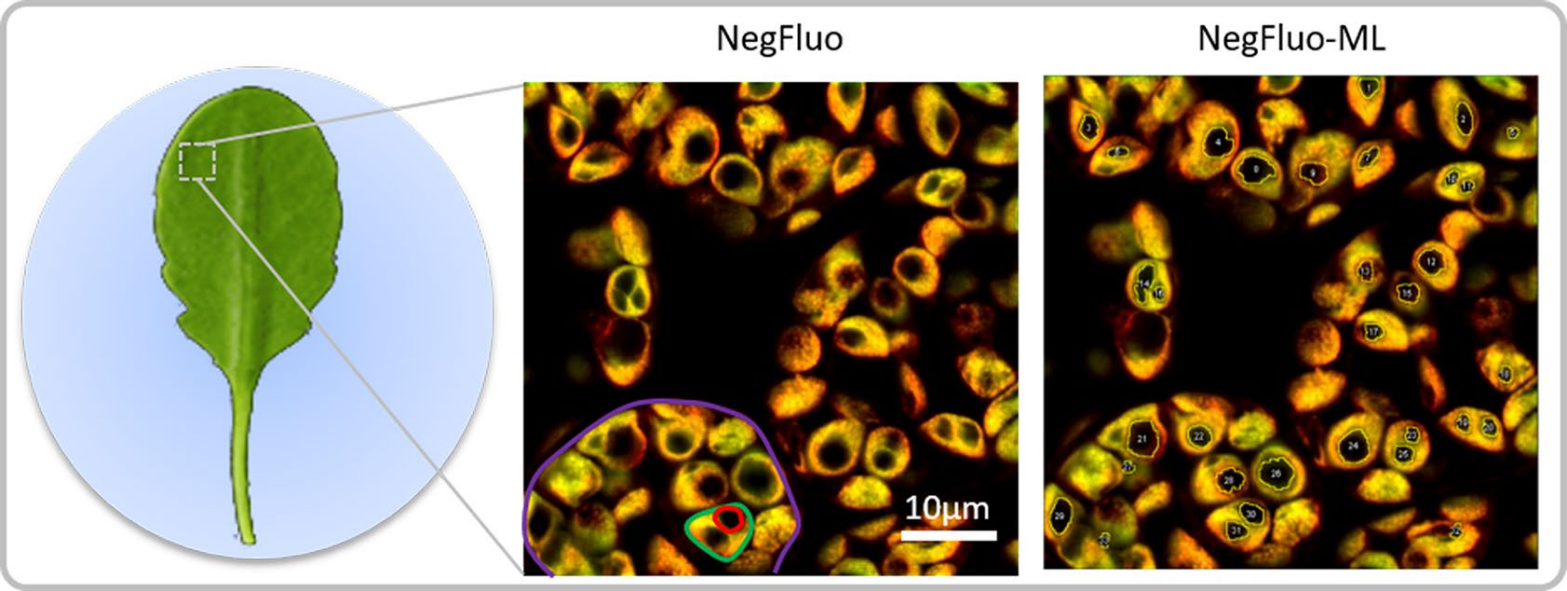
NegFluo starch analysis pipeline. Leaves of 2-week-old *Arabidopsis thaliana* were collected at the end of the illuminated period and fixed. A fragment measuring 2 × 2 mm was placed between the slide and coverslip before autofluorescence confocal imaging. In-focus cells, plastids, and starch granules can easily be observed as respectively highlighted in violet, green, and red through NegFluo. A local enhancement step is then applied to ease the machine learning-based segmentation step, NegFluo-ML. Starch granules size and morphology can then be measured.

Two color images were then processed using a homemade ImageJ macro in order to reduce the noise and to increase local contrast. This macro further improves contrast between starch granules and the surrounding plastids. In order to validate this new visualization method, we first chose two well-described and contrasted samples: a wild-type reference (Col-0) and the *ss4* mutant. *A. thaliana* leaves were collected at the end of the illuminated period (when the starch amount is maximum) and, for practical reasons, fixed with paraformaldehyde (**Figure 1**).

**Figure 2:**
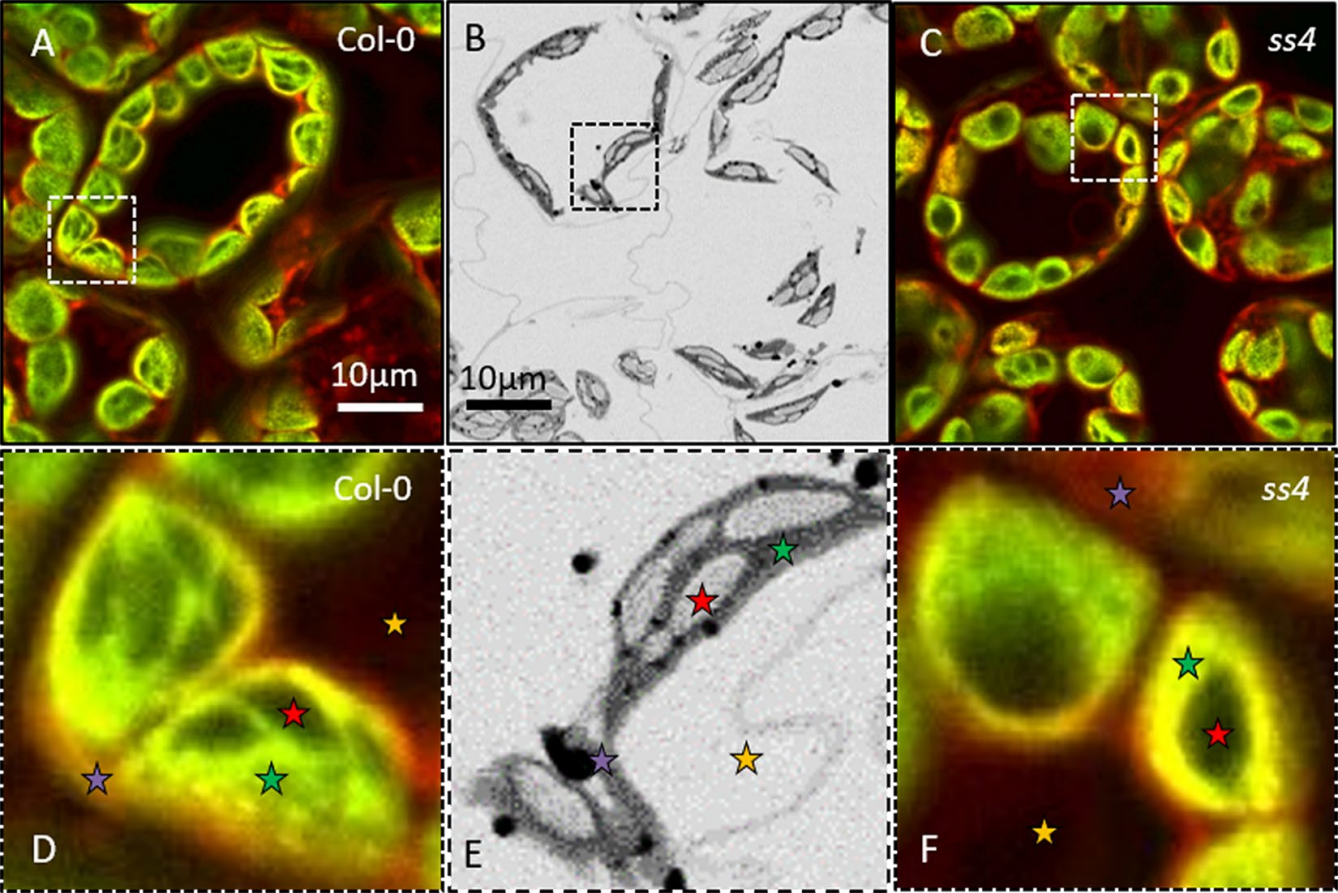
NegFluo applied to reference samples. To validate the procedure, we acquired NegFluo of Col-0 **(A** and **D)** and *ss4* **(C** and **F)** and ESEM images of Col-0 **(B** and **E)**. Both techniques provide the same morphological information. Representative leaf components are highlighted with colored stars (red, starch; green, plastid stroma; violet, cell membranes and cytosol; and yellow, background). Col-0, Columbia; ESEM, environmental scanning electron microscope.

These samples were then observed through NegFluo and compared with traditional electron microscopy imaging. As shown in **Figure 2**, both methods provide equivalent visual estimate of starch granule phenotype. We have recently applied NegFluo to decipher phenotypes of *pii1* mutant as described by Vandromme et al. (2019).

### Machine Learning for Automated Starch Granule Segmentation

To characterize the starch granules in more details, one needs to measure their size and shape. However, manual segmentation is time-consuming and not adapted to analyze a large number of starch granules. While NegFluo provides ease to visually analyze images, extracting robust parameters to automatically discriminate between the different structures of the leaf is not trivial using parametric approaches. We thus developed an ML-based pipeline to circumvent this technical limitation.

We developed the procedure based on Trainable Weka Segmentation module, since it combines the benefits of (i) FiJi (Schindelin et al., 2012) for image processing and simplicity of the manipulation of microscopy files by biologists and (ii) WEKA for ML (Frank et al., 2016).

Moreover, its implementation is modular and transparent, which allowed us to control the different steps as well as the possibility of customizing the algorithms and their evaluation (Arganda-Carreras et al., 2017). The NegFluo-ML algorithm follows two main steps: the extraction of images characteristics and the semantic segmentation by classification of pixels.

**Figure 3:**
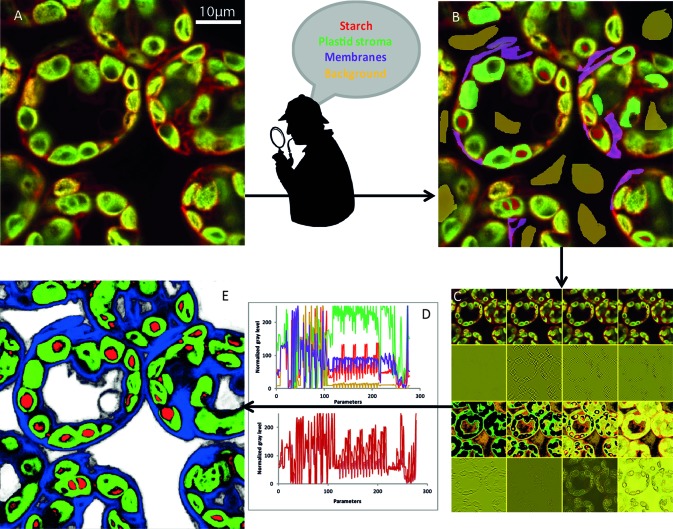
NegFluo-ML machine learning-based segmentation step. A set of NegFluo images **(A)** was manually and partially labeled in four classes **(B)** that were used for training (red, starch; green, plastid stroma; violet, membranes; and yellow, background). The algorithm then created from each image a stack of hundreds of its transformed versions (12 examples of the transformed image from **(A)** is presented in **(C)**, see text for parameters details). Thus, each pixel can now be described by hundreds of parameters than are used to discriminate between the four classes. In **(D)**, top graph, four pixels from different regions were compared (red, starch; green, plastid stroma; violet, cell membranes and cytosol; and yellow, background), while the bottom graph compares four pixels from starch region. The machine learning algorithm is then trained, using several images taken from different samples, to find the best sets of parameters allowing pixel classification between the four classes. Once the training reaches the desired accuracy level, it provides for each pixel a probability to be part of each class. It thus converts the original image into four probability maps **(E)** (red, starch; green, plastid stroma; blue, cell membranes and cytosol; and white, background). Finally, the trained algorithm is applied to a new set of images to validate its accuracy and ensure its applicability to any samples. Traditional segmentation algorithms can then be used for morphometric analysis.

(i) The initial image (**Figure 3A**) was first modified to highlight different characteristics of the objects. At first glance, we could classify them into four broad categories (Arganda-Carreras et al., 2017): edge detection, texture filters, noise reduction, and cytosol detection (**Figure 3B**). The set of these filters and the different parameters that we could adjust make it possible to obtain more than 300 images (**Figure 3C**) starting from the initial image. To achieve the best compromise between accuracy and training speed, we selected training features based on Gaussian blur, Sobel filter, Hessian, difference of Gaussians, membrane projections, variance, mean, anisotropic diffusion, bilateral, Lipschitz, Kuwahara, Gabor, entropy, and neighbors. The algorithm could then analyze each original pixel through 300 parameters that will be used to determine its belonging to the appropriate class (**Figure 3D**). The converted image was then generated based on attribution of each pixel to one of the different classes (**Figure 3E**).

(ii) Dozens of *Arabidopsis* leaf images were manually labeled in several classes. We tested configuration ranging from two classes (starch granules and non-starch granules) to five classes (starch granules, plastids, membranes, background, and vacuole). The optimal configuration consisted of defining four classes: the starch granule, the plastid, the cytosol delimited by cell membrane, and the background. Below four classes, the algorithm could not properly discriminate between starch granules and background, while five classes increased the number of pixels inaccurately attributed between these two dark-pixel structures. Different classifiers were also available. We selected the fast random forest (200 initial trees). It required a relatively low number of images for training (20 labeled images), was polyvalent, and avoided overfitting, and its computational cost for training was low. Part of the image pixels was then used for training, while the remaining pixels were subject to the algorithm to validate its accuracy (**Figure 3B**).

(iii) Once the classifier was trained, it was applied to new images and provided four probability maps. Thus, each pixel was assigned with a probability to be part of each category. Pixel color corresponds to the higher probability between the starch granule (red), the plastid (green), the membranes (blue), and the background (white) (**Figures 3D, E**).

(iv) From the probability maps, one could apply a traditional segmentation algorithm. To ensure optimal quality of starch detection, we developed an ImageJ macro (a) segmenting pixels with a probability higher than 80%, (b) segmenting starch granule with starch pixels embedded in plastids pixels, and (c) separating adjacent starch granules based on the watershed algorithm. Morphological properties of starch granules were then extracted from the segmented images (**Figure 3E**).

### Automated Morphological Characterization of Starch Granules

**Figure 4:**
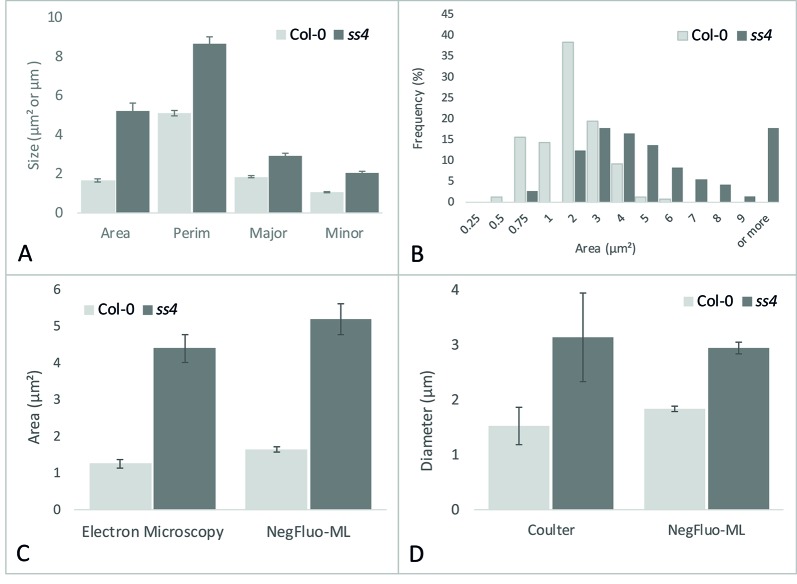
NegFluo-ML morphological analysis of Col-0 and *ss4* starch granules. Several parameters can be measured from segmented starch granules. In **(A)**, four parameters are represented: area (µm^2^), perimeter (Perim, µm), and major and minor axes (Major, Minor, µm). Beyond this traditional representation of means and standard error, this automated procedure ensures the analysis of hundreds of granules, allowing distribution analysis as depicted for granules area in **(B)**. NegFluo-ML thus allows unambiguous discrimination between Col-0 and *ss4* phenotypes. Furthermore, NegFluo-ML provides equivalent estimates for both area and diameter quantification compared with two state-of-the-art methods: manual quantification determined from transmission electron microscopy images **(C)** and Coulter counter **(D)**. For Coulter counter measurement, the values correspond to the average size of 30,000 particles determined from purified starch. The manual analysis of TEM images corresponds to a total of 61 and 70 granules, while NegFluo-ML automatically analyzed 153 and 74 granules for Col-0 and *ss4*, respectively. Vertical error bars represent a standard error. This value does not represent a measurement error but reflects the diversity of the starch granules sizes as illustrated in **(B)**. Col-0, Columbia.

While visual inspection of segmented images was perfectly consistent, NegFluo-ML allowed morphological characterization of starch granules, and various parameters such as area, perimeter, or diameter could be extracted (**Figure 4A**). In all cases, extracted parameters are significantly higher for *ss4* starch granules compared with wild type, which is in complete agreement with already published data (Roldán et al., 2007; Crumpton-Taylor et al., 2013). Furthermore, since the method was automated, a large number of granules could be measured, giving access to the global size distribution such as illustrated on the granules area in **Figure 4B**. In both Col-0 and *ss4* lines, starch granule size distribution behaves like a Gaussian curve. The curve is shifted toward higher size in the *ss4* line.

To ensure the relevance of these measurements, it was required to compare morphological parameters extracted from NegFluo-ML to state-of-the-art starch analysis methods. We thus analyzed images from Col-0 and *ss4* and compared them with both manual analysis of electron microscopy images (area, **Figure 4C**) and extracted starch granules analyzed with a Coulter counter (diameter, **Figure 4D**). For electron microscopy analyses, we used images previously acquired from TEM and manually determined starch granule size using ImageJ software. Several tens of starch granule areas were determined for Col-0 and *ss4* (61 and 70, respectively), while the average starch granule areas are significantly different between Col-0 and *ss4* using both methods (*p* < 0.001 with both techniques). Starch granules size was also determined using a Coulter counter, and the results were compared with those obtained by NegFluo-ML. Again, the results are consistent between the two methods, and the differences between the diameters of the Col-0 and *ss4* starch granules can be highlighted by both techniques

Measurements performed using NegFluo-ML were in perfect agreement with those obtained with previously used methods without the need to embed the samples in a resin for electron microscopy or to extract starch granules for Coulter analysis.

## Discussion

With this work, we propose a user friendly new pipeline for *in situ* starch granule morphological characterization. Previous works using confocal imaging for *in situ* starch granule observation (Ovecka et al., 2012) required several steps of sample preparation and iodine staining. In this example, starch granule is anti-correlated to chlorophyll autofluorescence signal. However, because of leaf autofluorescence heterogeneity, taking into account of chlorophyll autofluorescence alone does not allow precise discrimination of starch granule size and shape. The new proposed approach, combining imaging of several leaf autofluorescence signals, allows performing a negative fluorescence image providing the same morphological information without the need of starch granule staining or extensive sample preparation. It can be applied to both fresh and fixed leaf slices.

Although NegFluo provides an instant overview of starch granule phenotype, quantification of starch granule shape and size *in situ* remains challenging whatever the acquisition technique. We thus developed NegFluo-ML, a procedure combining state-of-the-art ML methods with segmentation methods from the traditional signal-processing field. While both methods are highly complementary, they overcome their individual limitations with a faster training, requiring only dozens of images and maintaining a high detection accuracy.

We validated these methods either by comparing them with high-throughput purified granule methods or by manual analysis of SEM images. While the results are highly comparable, NegFluo-ML presents several advantages. In NegFluo-ML compared with purified granule methods, morphological information can be associated to local information such as the number of granule per plastid and granule morphology, which are lost in a particle counter. Compared with manual analysis of leaf images, it provides two main advantages. The most obvious is the time gained through the process. Indeed, manual segmentation of images showing hundreds of starch granules is time-consuming and reduces, therefore, the overall number of analyzed structures. Furthermore, human segmentation is often highly impacted by user-biased analysis. Indeed, humans have their individual ways to select objects boundaries or to discard objects that seems nonrepresentative. Thus, different experimenters cannot perform such studies with a good reproducibility, and NegFluo-ML overcomes this limitation.

This manuscript focuses on the complete NegFluo/NegFluo-ML package applied to starch granule study in *Arabidopsis* leaf, but it can be extended. While both modules provide a complete acquisition and analysis pipeline, they are independent, and thus, NegFluo-ML can be transferred to other imaging methods such as SEM by adapting training features. Transposition to other plants could also be done after autofluorescence characterization adaptation. It thus opens the way to *in situ*, reliable, fast, and easy morphological characterization of starch granules.

## Data Availability

The datasets generated for this study are available on request to the corresponding author.

## Author Contributions

CV contributed to the investigation and formal analysis. AK, DT, and MF contributed to the formal analysis. AC and J-LP contributed to the investigation. CD’H contributed to the project administration, and manuscript review and editing. FW contributed to the conceptualization, methodology, and manuscript review and editing. CS contributed to the conceptualization, methodology, manuscript review and editing, and formal analysis.

## Funding

This work was supported by the CNRS (Centre National de la Recherche Scientifique) and the University of Lille. We thank Agence Nationale de la Recherche for funding (“CaSta-DivA” Project, grant no. ANR-11-BSV6-0003).

## Conflict of Interest Statement

The authors declare that the research was conducted in the absence of any commercial or financial relationships that could be construed as a potential conflict of interest.
